# Task-Specific Detector Adaptation for Edge MOT: Tracking and Deployment Trade-Offs on the NVIDIA Jetson Nano

**DOI:** 10.3390/jimaging12090411

**Published:** 2026-09-01

**Authors:** Bruna de Vargas Guterres, Juan Pedro de León, Pablo D. Cuña, Víctor Castelli, Silvia Silva da Costa Botelho, Marcelo Rita Pias

**Affiliations:** 1 Postgraduate Program on Robotics and Artificial Intelligence, Technological University of Uruguay (UTEC), Rivera 40000, Uruguay; juan.deleon@utec.edu.uy (J.P.d.L.); pablo.cuna@utec.edu.uy (P.D.C.); victor.castelli@utec.edu.uy (V.C.); 2 Computer Science Center, Federal University of Rio Grande (FURG), Rio Grande 96203-900, RS, Brazil; silviacb@furg.br (S.S.d.C.B.); mpias@furg.br (M.R.P.)

**Keywords:** edge AI, MOT, embedded systems

## Abstract

Although several MOT solutions have been proposed, limited evidence is available regarding how task-specific detector adaptation affects tracking quality and deployment requirements on low-cost edge hardware. These effects have not been jointly evaluated under fixed tracking and deployment conditions. This work evaluates MOT on a 4 GB NVIDIA Jetson Nano using the MOT17 benchmark. Experiment 1 served as a baseline characterization and detector-selection stage. YOLOv8n, SSDLite320 and Faster R-CNN were evaluated with a fixed OC-SORT configuration under the same tracking framework. Based on the observed trade-offs, YOLOv8 was selected for adaptation. A task-specific fine-tuning stage was subsequently performed for YOLOv8n and YOLOv8s using more than 30,000 annotated pedestrian image records. Experiment 2 constituted the main analysis. Baseline and adapted models were evaluated under the same tracking and deployment protocol. Tracking performance was assessed through MOTA, IDF1, and HOTA. Processing throughput was measured together with system RAM usage. Board power consumption and energy per frame were also measured. In the paired YOLOv8n comparison, MOTA increased from 15.95 to 51.09 after adaptation. Throughput changed from 3.6 to 3.5 FPS. System RAM usage changed from 3.3820 to 3.3925 GB. Average board power changed from 4235 to 4227 mW. Energy per frame increased by approximately 2.7%. The adapted YOLOv8s model achieved a MOTA of 55.88 at 2.5 FPS. None of the evaluated pipelines achieved conventional real-time video throughput. These findings indicate that task-specific adaptation improved tracking performance with limited changes in the measured deployment characteristics within the evaluated configuration.

## 1. Introduction

Multiple Object Tracking (MOT) has become a fundamental component of modern vision systems used in intelligent surveillance, autonomous vehicles, robotics, and smart city applications. In these scenarios, objects must not only be detected but also consistently identified across consecutive frames to allow motion analysis, trajectory estimation, and decision making. Recent advances in deep learning have improved tracking performance on public benchmarks, leading to the adoption of increasingly sophisticated detector and tracker architectures [1,2].

Most embedded MOT systems continue to rely on the tracking-by-detection paradigm, where an object detector first localizes targets and an association stage subsequently links detections across frames [3,4,5]. Alternative approaches have attempted to reduce computational requirements through joint detection and embedding architectures or adaptive detection strategies that execute detection only when required [5,6,7]. Despite these advances, tracking introduces additional processing requirements beyond object detection. Motion estimation, similarity computation, appearance feature extraction, trajectory management, and data association contribute additional computational overhead that may become relevant in resource-constrained environments [1,2,8].

Recent studies have demonstrated that MOT systems can be deployed on modern edge hardware. HopTrack achieved 39.29 FPS and 63.12% MOTA on the MOT16 benchmark using an NVIDIA Jetson AGX Xavier platform [6], while FPGA-based solutions have also reported real-time operation through detector quantization and hardware-specific optimizations [2,3]. Embedded deployments have been reported on NVIDIA Jetson devices, Qualcomm platforms, heterogeneous systems on chip, low-cost edge AI boards, and reconfigurable hardware architectures [4,9,10,11]. These studies demonstrate that real-time MOT has been achieved on selected embedded platforms under specific hardware and optimization conditions. However, the reported performance depends on the evaluated workload and deployment configuration.

Although object detection models have been extensively evaluated on embedded devices, considerably less attention has been devoted to the behavior of complete detector tracking pipelines under deployment constraints. In particular, limited evidence is available regarding the trade-offs between tracking quality, execution speed, memory usage, and energy consumption when popular tracking-by-detection systems are deployed on low-cost edge hardware. Practical studies have also shown that detector configuration choices, confidence thresholds, and processing resolution can substantially alter tracking performance and ranking outcomes across benchmarks [12]. As a result, selecting a detector tracking configuration for embedded deployment remains a task that depends on application-specific requirements and hardware limitations.

The NVIDIA Jetson Nano remains one of the most accessible embedded AI platforms adopted in research and education. Although more recent embedded GPUs provide higher computational capability, the Jetson Nano continues to represent an important reference platform for low-cost edge deployment.

This work investigates task-specific detector adaptation for MOT deployment on a 4 GB NVIDIA Jetson Nano. Experiment 1 evaluates YOLOv8n, Faster R-CNN and SSDLite320 under a fixed OC-SORT configuration and tracking framework. Based on the observed trade-offs, YOLOv8 is selected for adaptation. Experiment 2 compares baseline and adapted YOLOv8 models under the same protocol. Tracking quality is assessed using standardized MOT metrics. Processing throughput and system RAM usage are measured. Board power consumption and energy per frame are also reported. This study is guided by the following research questions:

- RQ1: What tracking and deployment trade-offs are observed among representative detector configurations under a fixed OC-SORT pipeline on the NVIDIA Jetson Nano?
- RQ2: How does task-specific adaptation of the selected detector affect tracking performance and deployment requirements under the same experimental protocol?

The contribution of this study is empirical. Task-specific detector adaptation is examined within an existing MOT pipeline under fixed tracking and deployment conditions. The study provides the following contributions:

- A paired evaluation of baseline and task-specific YOLOv8n models under the same OC-SORT configuration. The detector architecture and deployment protocol were maintained throughout this comparison.
- A controlled system-level comparison of representative detectors under a fixed OC-SORT configuration on the NVIDIA Jetson Nano.
- An analysis of tracking quality and deployment requirements after detector adaptation. Throughput and memory use are measured. Power consumption and energy per frame are also reported.

## 2. Related Work

Recent studies have demonstrated that Multiple Object Tracking (MOT) can be deployed on embedded computing platforms through lightweight detector tracker pipelines and hardware-specific optimizations. Representative implementations have been reported on NVIDIA embedded GPUs and FPGA-based systems [2,6]. These studies show that real-time operation has been achieved on selected platforms under specific optimization conditions. End-to-end studies also indicate that deployment performance depends on the complete processing pipeline rather than tracker execution alone. Detector execution and image preprocessing contribute to the total computational cost. Hardware limitations also affect the observed throughput [12].

Most embedded MOT systems adopt the tracking-by-detection paradigm, where object detection is first performed and detections are subsequently associated across consecutive frames [3,5]. Under this framework, detector quality directly affects tracking performance because missed detections and localization errors propagate to the association stage [13,14]. Studies of embedded MOT identify detector execution as a main source of computational cost within the evaluated pipelines. Detector configuration also influences tracking accuracy and deployment performance [1,2,6].

Representative detector architectures exhibit different computational profiles. One-stage detectors, including You Only Look Once (YOLO) [15] and Single Shot MultiBox Detector (SSD) [16], perform object localization and classification during a single forward pass. These architectures are therefore widely adopted for real-time applications when lower inference latency is required. Two-stage detectors such as Faster R-CNN [17] first generate region proposals and subsequently classify candidate objects. This design generally improves localization accuracy at the expense of increased computational complexity [18,19]. Existing benchmark studies also report that detector settings, including confidence thresholds and input resolution, influence both tracking quality and computational requirements [12].

Tracking algorithms differ in the mechanisms used to associate detections across consecutive frames. Simple Online and Real-time Tracking (SORT) introduced a lightweight online tracking framework based on Kalman filtering and Hungarian assignment [20]. Observation Centric SORT (OC-SORT) extends this formulation through observation centric motion estimation while preserving the tracking-by-detection paradigm [21,22]. OC-SORT does not require an appearance-based re-identification stage. This design avoids the computational requirements associated with extracting and comparing appearance features. It is therefore relevant to MOT deployment under constrained computational resources.

Detector adaptation has also been examined as a strategy for improving tracking-by-detection pipelines. Xiao et al. [13] combined an improved YOLOv8 detector with OC-SORT for pedestrian tracking. Their results showed that changes to the detection stage affected the resulting tracking metrics. Alikhanov et al. [12] also showed that detector configuration and evaluation settings can alter MOT performance and model rankings. However, these studies did not jointly examine task-specific detector adaptation and device-level deployment requirements through a paired baseline and adapted comparison on low-cost embedded hardware.

The present study addresses this gap through a paired comparison of baseline and adapted YOLOv8n models. The detector architecture and OC-SORT configuration were maintained across this comparison. The hardware platform and evaluation benchmark were also unchanged. Deployment measurements were collected under the same protocol.

## 3. Materials and Methods

**Figure 1:**
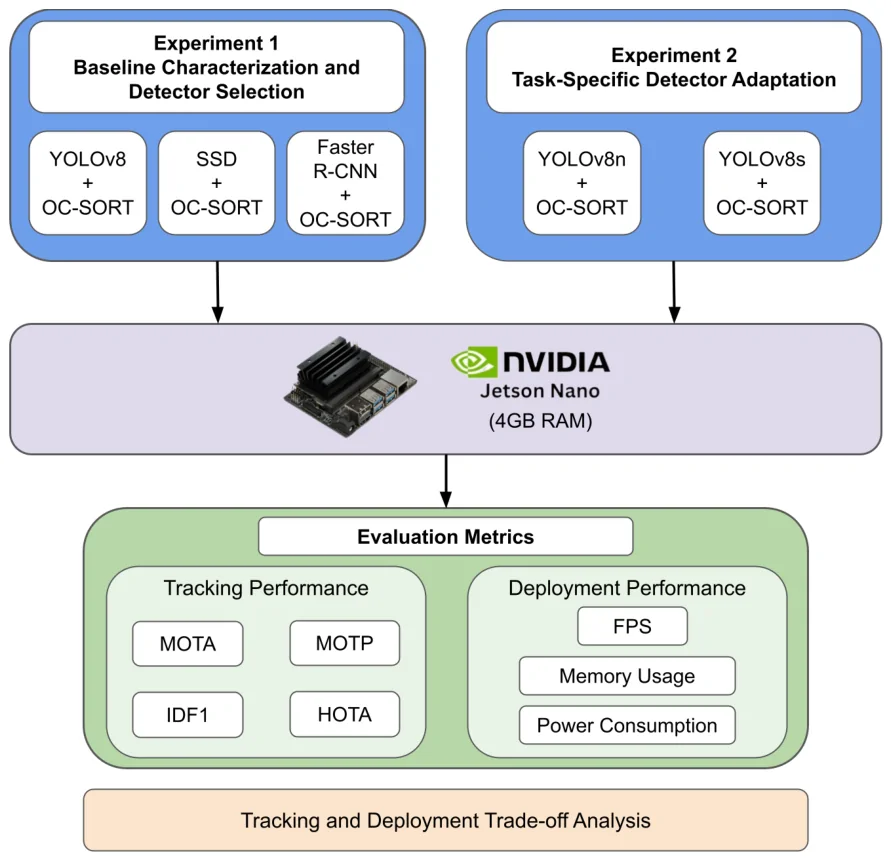
Experimental protocol used to evaluate task-specific detector adaptation after baseline characterization and detector selection.

Figure 1 summarizes the experimental workflow described in the following subsections.

### 3.1. Experimental Design

The experimental protocol comprised two complementary experiments conducted under identical hardware and evaluation conditions.

Experiment 1 (Baseline Characterization and Detector Selection) served as a baseline characterization and detector-selection stage. Representative detector configurations were compared under a fixed OC-SORT pipeline using the MOT17 benchmark dataset [23]. The YOLOv8, SSD, and Faster R-CNN detector architectures were evaluated using pretrained weights from the Microsoft COCO dataset. The same OC-SORT MOT was maintained, allowing detector performance to be analyzed independently of tracker behavior.

Experiment 2 (Task-Specific Detector Adaptation) constituted the main analysis. It evaluated how detector adaptation affected tracking and deployment metrics. YOLOv8 model family was selected for adaptation based on the tracking and deployment trade-offs observed during Experiment 1. YOLOv8n and YOLOv8s models were fine-tuned using the Merged Pedestrian Dataset. They were subsequently evaluated on the MOT17 benchmark dataset [23] under the same hardware conditions, OC-SORT setup and deployment protocol. The main characteristics of both experiments are summarized in Table 1.

### 3.2. Experimental Platform and Inference Configuration

All experiments were conducted on an NVIDIA Jetson Nano equipped with 4 GB of RAM. This platform was selected because it represents a low-cost embedded computing device commonly adopted for edge AI and computer vision applications. It provides limited computational resources, memory capacity, and power availability for characterizing complete MOT pipelines under fixed memory and power constraints.

The NVIDIA Jetson Nano provides a 10 W MAXN profile (mode 0) and a 5 W profile (mode 1). All experiments used the 10 W MAXN profile. The power profile was not varied. The initial swap allocation was increased from 2 GB to 4 GB before the experiments. This configuration was maintained throughout all evaluations.

The system ran Ubuntu Focal 20.04.3 LTS with Python 3.8.10 and Ultralytics 8.0. PyTorch version 1.8 provided the execution framework. All detector configurations used the Jetson Nano GPU through the CUDA backend. Detector inference was performed using FP32 precision. CPU-only detector inference was not used. The *jetson_clocks* utility was not enabled. The default dynamic clock behavior was therefore retained. The reported throughput characterizes the evaluated PyTorch configurations without hardware-specific optimizations (e.g., TensorRT conversion, FP16 and INT8 quantization, structured pruning).

The inference applications were launched through the command line. Graphical visualization was disabled and the Ubuntu desktop environment remained active under the same conditions throughout all experiments. Board power consumption and physical RAM utilization were monitored using Jetson Stats (jtop) [24]. Consequently, system-level measurements include the background resource utilization associated with the desktop environment.

The original MOT17 frames were processed using detector-specific input transformations. YOLOv8n and YOLOv8s used an inference target of 640×640 pixels. The aspect ratio was preserved through internal resizing and padding. SSDLite320 used its fixed input resolution of 320×320 pixels. Faster R-CNN preserved the image aspect ratio while resizing the shorter side to 800 pixels. The longer side was limited to 1333 pixels. Consequently, Experiment 1 characterizes complete deployment configurations rather than detector architectures under a common input resolution.

Detector adaptation was performed at a training resolution of 1280×1280 pixels. Baseline and adapted YOLOv8 models used the same 640×640 inference target during Experiment 2. Therefore, no inference-resolution difference was introduced into this comparison.

Table 2 presents the detector confidence thresholds and Non-Maximum Suppression (NMS) settings. NMS used Intersection over Union (IoU) thresholds to remove overlapping predictions. The Faster R-CNN settings distinguish between the Region Proposal Network (RPN) and the final predicted boxes. Confidence and NMS thresholds were not manually tuned. The defaults of the corresponding inference frameworks were retained. OC-SORT used a threshold value of 0.30 across all configurations.

### 3.3. Datasets

Two datasets with distinct purposes were employed in this study. The MOT17 benchmark [23] was adopted as the reference dataset for evaluating all detector tracking pipelines during both experiments. A second dataset, referred to as the Merged Pedestrian Dataset, was assembled to perform task-specific detector adaptation during Experiment 2.

MOT17 [23] is one of the most widely adopted benchmarks for MOT research. It comprises annotated urban video sequences containing pedestrian trajectories acquired under diverse environmental conditions. The benchmark provides standardized annotations and evaluation protocols, enabling objective and reproducible comparisons between different MOT pipelines. The same MOT17 [23] test split was used to evaluate the baseline and adapted detector configurations. Only pedestrian annotations were considered, since pedestrian tracking represents the target application of the evaluated MOT pipelines.

The Merged Pedestrian Dataset was constructed by combining well-established pedestrian datasets from the MOTChallenge initiative, including MOT16 and MOT20 [23,25], with complementary publicly available pedestrian datasets, namely Person-mfa1g [26]. This strategy increased the diversity of pedestrian appearances, viewpoints, object scales, and scene conditions available during detector adaptation while preserving a common target class across all datasets.

Before merging, all annotations were converted to the COCO format to provide a unified annotation structure. Only the person class was retained during dataset preparation, since pedestrian detection represents the target application of the evaluated MOT pipelines. The resulting Merged Pedestrian Dataset comprised more than 30,000 annotated images, which were divided into 27,000 images for training and 3000 images for validation. Table 3 summarizes the datasets employed in each experiment.

An audit of the fine-tuning and evaluation data was conducted after dataset assembly. Dataset provenance and file structure were reviewed. Image content was also visually inspected. The fine-tuning data included images from the MOT16 and MOT17 training sequences 02, 04, 05, 09, 10, 11, and 13. Evaluation used the MOT17 test sequences 01, 03, 06, 07, 08, 12, and 14. None of these test-sequence identifiers was present in the fine-tuning directories.

The MOT20 and Person-mfa1g components were reviewed separately. No image matching a scene or frame from the MOT17 test split was identified. Person-mfa1g contains pedestrian images from contexts distinct from the evaluated MOTChallenge sequences. These images include rural and recreational contexts. Transportation and indoor scenes are also represented. No scene-level or sequence-level overlap with the MOT17 test split was identified.

### 3.4. Evaluated MOT Pipelines

The OC-SORT tracker was selected because it provides a lightweight online tracking framework suitable for real-time edge deployment while avoiding the additional computational cost associated with appearance-based re-identification methods. By maintaining a fixed tracker configuration, the influence of the object detector on both tracking performance and deployment requirements could be evaluated under identical conditions.

The detector set was defined to provide representative computational profiles rather than an exhaustive comparison of edge-oriented detectors. Selection required pretrained COCO weights, implementation within the adopted PyTorch environment, integration with the detection interface used by OC-SORT, and execution under the same Jetson Nano protocol. Three detector architectures with distinct computational characteristics were selected for the baseline evaluation. YOLOv8 represents a modern one-stage detector widely adopted for real-time computer vision applications. SSD was included as a lightweight single-shot detector designed for high-throughput inference. Faster R-CNN was selected as a two-stage detector representing higher detection capacity at the expense of increased computational complexity. Together, these detectors provide representative deployment profiles ranging from lightweight to computationally demanding architectures.

YOLOv8n was loaded from the Ultralytics framework. SSDLite320 was implemented using the MobileNetV3-Large as backbone. Faster R-CNN was implemented using a ResNet-50 backbone with a Feature Pyramid Network.

### 3.5. Evaluation Metrics

The detector configurations evaluated in this study were assessed using complementary tracking and deployment metrics. Tracking performance was measured using the standardized MOTChallenge evaluation protocol. Multiple Object Tracking Accuracy (MOTA) and Multiple Object Tracking Precision (MOTP) were reported. Identification Precision (IDP) and Identification Recall (IDR) were also calculated. The Identification F1 Score (IDF1) was used to summarize identity preservation. Higher Order Tracking Accuracy (HOTA) was used to jointly characterize detection and association performance [27,28].

Deployment characteristics were evaluated through processing throughput, memory usage, power consumption, and energy efficiency measured on the NVIDIA Jetson Nano. System-level power consumption and physical RAM usage were monitored using Jetson Stats (jtop). Power was obtained from the tot field, which corresponds to total board consumption rather than the isolated consumption of the detector or GPU. Physical RAM usage corresponds to the total system RAM use rather than the detector-specific memory footprint.

Processing throughput was quantified as Frames-Per-Second (FPS), while average system-level memory usage and average power consumption characterized the computational requirements associated with each detector configuration. Energy efficiency was additionally characterized through the energy required to process a single video frame, reported as Energy per Frame (mJ/frame). This metric was estimated by dividing the average power consumption by the processing throughput (FPS):

(1) $$\mathrm{EPF}=\frac{P_{\mathrm{avg}}}{\mathrm{FPS}},$$

where EPF denotes the Energy Per Frame, expressed in millijoules per frame (mJ/frame), and $P_{\mathrm{avg}}$ represents the average power consumption measured during inference. Lower values indicate more energy efficient detector configurations.

The reported throughput values represent average processing performance across the evaluated frames. Frames were not stratified according to the number of ground-truth pedestrians, retained detections, or active tracks. Consequently, the measurements characterize the average workload of each evaluated configuration rather than its performance at specific levels of scene density.

Controlled repeated deployment trials were not conducted. Dispersion estimates were therefore not calculated. The same evaluation protocol was adopted throughout both experiments to enable a direct comparison between the baseline and adapted detector configurations.

### 3.6. Task-Specific Detector Adaptation

Experiment 2 investigated whether task-specific detector adaptation could improve MOT deployment performance while preserving the computational characteristics required for edge deployment. Based on the baseline evaluation conducted in Experiment 1, the YOLOv8 detector was selected for adaptation because it provided the most favorable balance between tracking performance and deployment requirements. Two detector variants, YOLOv8n and YOLOv8s, were independently adapted using the Merged Pedestrian Dataset described in Section 3.3.

Detector adaptation was performed using the Ultralytics YOLOv8 framework. Both models were initialized from the corresponding pretrained weights provided by Ultralytics and subsequently fine-tuned using the Merged Pedestrian Dataset. Due to the computational limitations of the NVIDIA Jetson Nano, detector training was performed on a desktop workstation equipped with an Intel Core i7-7700K processor, 32 GB DDR4 RAM, an NVIDIA RTX 2080 GPU with 12 GB of memory, and Ubuntu Desktop 22.04 LTS.

Training was conducted for 200 epochs using a batch size of 16 images and an input resolution of 1280×1280 pixels. Model performance was monitored throughout training using the validation set, and the best performing model was retained for subsequent evaluation.

The adapted detectors were integrated into the same OC-SORT tracking pipeline adopted during Experiment 1. They were evaluated on the MOT17 benchmark using the identical deployment protocol and evaluation metrics. This procedure enabled a direct comparison between the baseline and adapted detector configurations while keeping all remaining experimental conditions unchanged.

## 4. Results

This section presents the results obtained from the two experiments described in Section 3. For both experiments, tracking performance and deployment characteristics are presented separately to facilitate the analysis of detector trade-offs.

### 4.1. Experiment 1—Baseline Characterization and Detector Selection

#### 4.1.1. Tracking Performance—Experiment 1

**Figure 2:**
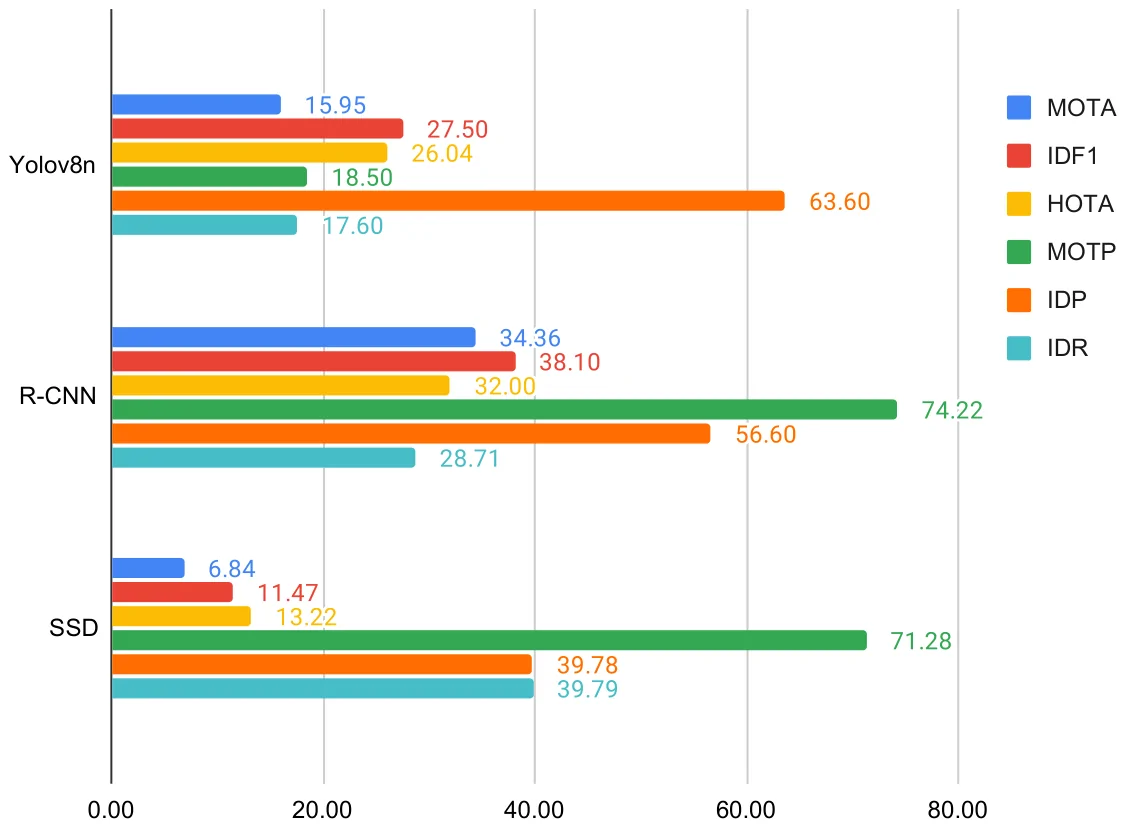
Comparison of tracking performance obtained by the evaluated detector configurations during Experiment 1. Evaluation metrics include MOTA, IDF1, HOTA, MOTP, IDP, and IDR.

Figure 2 presents a graphical comparison of the tracking metrics obtained by the evaluated detector configurations. The same ranking was observed for MOTA, IDF1, and HOTA.

Faster R-CNN achieved the highest MOTA, IDF1, HOTA, and MOTP values, reaching a MOTA of 34.36, an IDF1 score of 38.10, and a HOTA score of 32.00. YOLOv8n obtained intermediate MOTA, IDF1, and HOTA values and achieved the highest IDP at 63.60. SSD presented the lowest MOTA, IDF1, HOTA, and IDP values. However, its MOTP of 71.28 was close to the 74.22 obtained by Faster R-CNN, and it achieved the highest IDR at 39.79. Therefore, the evaluated detectors did not follow a uniform ranking across all tracking metrics.

#### 4.1.2. Deployment Performance—Experiment 1

Table 4 summarizes the deployment metrics obtained during Experiment 1 according to processing throughput, average power consumption, memory utilization, and estimated Energy per Frame (EPF). SSD achieved the highest processing throughput, reaching 5.0 FPS, followed by YOLOv8n with 3.6 FPS, whereas Faster R-CNN processed the benchmark at only 0.4 FPS. In contrast, average power consumption remained nearly constant across all detector configurations, varying from 4201 mW to 4252 mW. Similarly, average memory utilization remained close to 3.38 GB for all evaluated detectors. Throughput ranged from 0.4 to 5.0 FPS across the baseline configurations. These values remained below conventional real-time video rates.

The narrow range of system RAM usage represents total physical RAM use during execution of the complete MOT pipeline. All configurations shared the operating system and active desktop environment. The Python and PyTorch/CUDA runtimes were also common. OC-SORT and the processing buffers were maintained across configurations. These shared components contributed to a common system memory baseline. The reported values therefore characterize total platform memory occupancy rather than detector-specific memory use.

Although average power consumption showed only marginal variation, the estimated EPF differed substantially among detector configurations. SSD achieved the lowest EPF (840.2 mJ/frame), followed by YOLOv8n (1176.4 mJ/frame), whereas Faster R-CNN required 10,630.0 mJ/frame. These results indicate that the large differences in deployment efficiency were primarily associated with processing throughput rather than instantaneous power consumption.

YOLOv8 provided an intermediate profile between tracking quality and processing throughput. This result motivated its selection for task-specific adaptation in Experiment 2.

### 4.2. Experiment 2—Task-Specific Detector Adaptation

#### 4.2.1. Tracking Performance—Experiment 2

**Figure 3:**
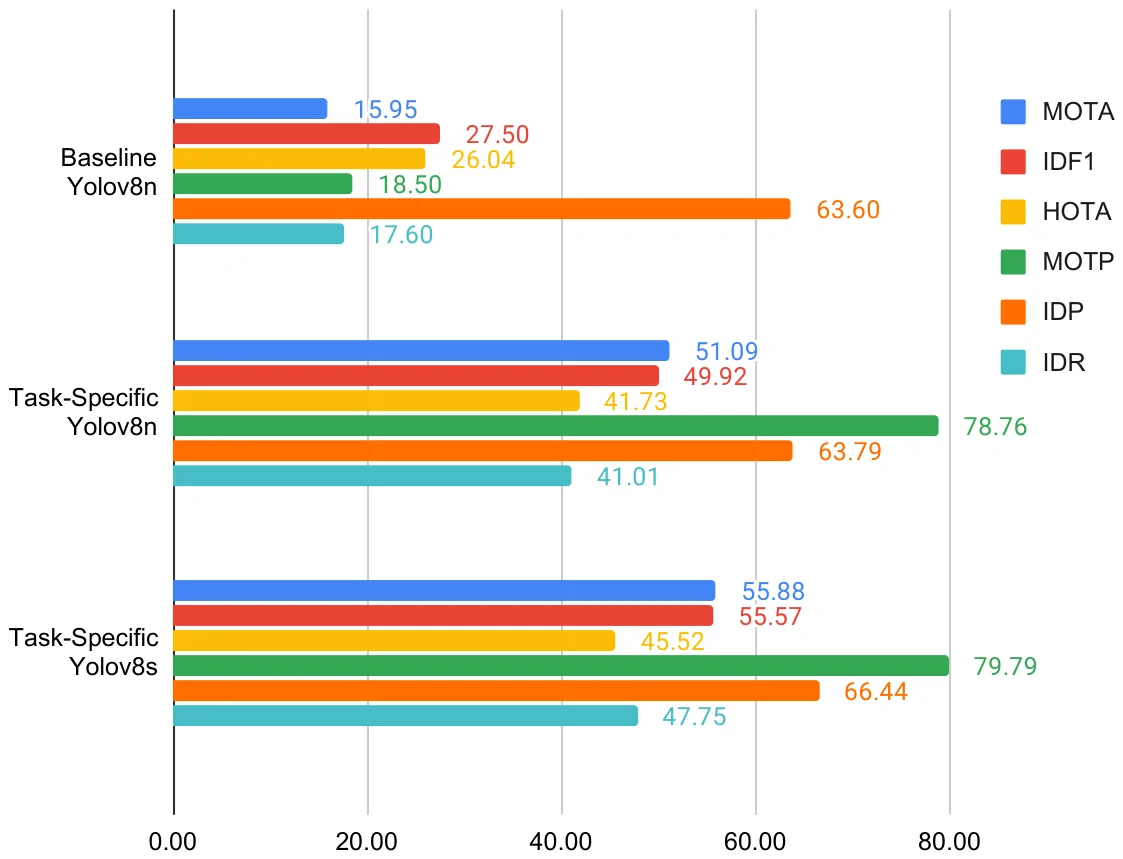
Comparison of tracking performance obtained by the evaluated detector configurations during Experiment 2. Evaluation metrics include MOTA, IDF1, HOTA, MOTP, IDP, and IDR.

Figure 3 compares the baseline detector with two task-specific adapted versions. Within the reported evaluation, the adapted configurations produced higher MOTA, IDF1, HOTA, MOTP, and IDR values than the baseline YOLOv8n configuration. MOTA increased from 15.95 for baseline YOLOv8n to 51.09 and 55.88 for adapted YOLOv8n and YOLOv8s, respectively.

IDP remained similar across the evaluated configurations. Higher MOTP and IDR values were observed after adaptation. The adapted YOLOv8s configuration produced the highest observed values for most tracking metrics.

#### 4.2.2. Deployment Performance—Experiment 2

Table 5 summarizes the deployment metrics obtained after task-specific detector adaptation according to processing throughput, average power consumption, system-level memory usage, and estimated Energy per Frame (EPF).

The paired YOLOv8n comparison provides the direct assessment of task-specific detector adaptation. The estimated EPF increased from 1176.39 to 1207.71 mJ/frame. This difference represents an increase of approximately 2.7% in the energy required to process each frame. The adapted YOLOv8n detector maintained processing throughput comparable to the baseline configuration, decreasing slightly from 3.6 to 3.5 FPS. Given the unchanged detector architecture and inference settings, the 0.1 FPS difference is interpreted as a small variation in the system-level measurement.

The task-specific YOLOv8s configuration processed the benchmark at 2.5 FPS, while its board power and system-level RAM usage remained within the narrow ranges observed for the YOLOv8n configurations. Its EPF reached 1688.40 mJ/frame, primarily reflecting its lower processing throughput.

## 5. Discussion

RQ1: What tracking and deployment trade-offs are observed among representative detector configurations under a fixed OC-SORT pipeline on the NVIDIA Jetson Nano?

Experiment 1 demonstrated that detector selection affected both tracking quality and deployment efficiency. Faster R-CNN achieved the highest MOTA, IDF1, HOTA, and MOTP values, whereas SSD provided the highest processing throughput and the lowest estimated Energy per Frame (EPF). Average board-level power consumption and system-level RAM usage varied within narrow ranges across the evaluated configurations. EPF differed by more than one order of magnitude, indicating that processing latency rather than instantaneous power demand dominated the energy cost of deployment.

This behavior is consistent with previous studies showing that most embedded MOT systems continue to adopt the tracking-by-detection paradigm, where tracking quality depends directly on detector performance [3,5]. Under this framework, localization errors and missed detections propagate to the association stage and reduce overall tracking quality [13,14]. Previous work has also identified detector execution as the dominant source of computational cost in embedded MOT systems [2,6].

Low single-digit throughput has also been reported on the 4 GB Jetson Nano under native PyTorch inference. Nnadozie et al. [29] reported processing rates between 5.6 and 7.0 FPS for native PyTorch YOLOv5 configurations on this platform. The corresponding TensorRT configurations achieved between 8.1 and 10.5 FPS. Although that study evaluated object detection rather than a complete MOT pipeline, its measurements provide relevant context for the 2.5–3.6 FPS obtained by the YOLOv8–OC-SORT configurations in the present study. Direct numerical comparison remains limited by differences in detector architecture, input resolution, dataset, runtime, and pipeline composition.

Lema et al. [30] provided a broader characterization of YOLO deployment on the NVIDIA Jetson Nano. Eleven configurations from the YOLOv5, YOLOX, and YOLOv3 families were evaluated at 20 input sizes ranging from 320 to 1216 pixels using PyTorch and TensorRT. At an input size of 640 pixels, the reported PyTorch inference times correspond to approximately 13.8, 7.6, 3.3, and 1.9 FPS for YOLOv5-N, YOLOv5-S, YOLOv5-M, and YOLOv5-L, respectively. The corresponding TensorRT measurements were approximately 16.2, 11.2, 5.3, and 2.9 FPS. The authors reported no change in AP between the PyTorch and TensorRT configurations, although some larger models could not be evaluated because of memory limitations.

The analysis by Lema et al. also showed that the 30 FPS criterion was reached only by simpler models operating at reduced input sizes with TensorRT. Suo et al. [31] similarly reported that a combined optimization strategy increased YOLOv3 throughput from 3.9 to 13.1 FPS. These findings show that model complexity, input resolution, runtime format, and numerical precision affect embedded inference throughput.

Several native PyTorch configurations evaluated by Lema et al. produced throughput values of the same order as those measured in the present study. However, their measurements represented detector inference, whereas the present measurements represent the complete detector-OC-SORT pipeline. The evaluated pipeline also performs detection handling, data association, and track-state updates for each frame. These operations introduce additional computational overhead relative to detector-only inference. TensorRT conversion, reduced-precision inference, and model pruning were not applied in the present study. The reported results therefore characterize the evaluated FP32 PyTorch pipelines rather than hardware-optimized deployment configurations.

The present results complement these observations by comparing representative detector configurations under a common tracking framework on a low-cost embedded platform. Unlike studies conducted on higher-performance hardware, such as the Jetson AGX Xavier [6], the evaluation performed on the Jetson Nano illustrates how detector complexity constrains deployment on a platform with limited computational resources. The results indicate that detector selection should be guided jointly by tracking quality and deployment efficiency. Evaluating tracking metrics alone may overlook substantial differences in computational cost, whereas considering only throughput ignores important differences in tracking performance.

RQ2: How does task-specific adaptation of the selected detector affect tracking performance and deployment requirements under the same experimental protocol?

Experiment 2 demonstrated that task-specific detector adaptation increased the reported tracking metrics while preserving similar system-level RAM utilization and average board power consumption. In the paired YOLOv8n comparison, MOTA increased from 15.95 to 51.09, corresponding to an increase of 35.14 percentage points. Processing throughput changed from 3.6 to 3.5 FPS, while EPF increased by approximately 2.7%. The adapted YOLOv8s configuration reached a MOTA of 55.88 at 2.5 FPS, showing an additional trade-off between tracking performance and processing throughput.

The observed increases in the tracking metrics are consistent with previous studies reporting that detector quality directly affects tracking performance in tracking-by-detection systems [1,12]. Improvements in pedestrian localization reduce missed detections and false positives before the association stage, allowing the tracker to maintain more consistent trajectories. Xiao et al. [13] combined an improved YOLOv8 detector with OC-SORT for pedestrian tracking. Their results showed that modifications to the detection stage affected the performance of the complete tracking pipeline. However, their approach modified the detector architecture. The paired comparison in the present study retained the YOLOv8n architecture and examined task-specific training under fixed deployment conditions.

OC-SORT processed the outputs generated by each detector configuration. Differences in missed and false detections therefore affected the observations and candidate detections provided to the same association procedure. The resulting metrics characterize the performance of the complete detector-OC-SORT configurations rather than changes to the intrinsic OC-SORT algorithm.

Alikhanov et al. [12] showed that confidence thresholds and processing resolution can affect tracking metrics and configuration rankings. In the present paired comparison, the detector architecture, tracker, evaluation benchmark, inference settings, and deployment protocol were maintained. This design reduces the influence of configuration changes when examining detector adaptation. The analysis also extends beyond tracking metrics by considering throughput, system-level RAM usage, average board power consumption, and EPF.

Previous embedded MOT studies have primarily focused on detector architecture or tracker design [2,6]. Comparatively fewer studies have investigated the effect of task-specific detector adaptation on deployment trade-offs. The present study addresses this gap through a paired comparison of baseline and adapted YOLOv8n models within an existing modular pipeline. The runtime format, numerical precision, detector architecture, and OC-SORT configuration were maintained across this comparison. The observed differences therefore describe task-specific adaptation under the evaluated PyTorch deployment conditions rather than hardware-specific execution acceleration.

### 5.1. Practical Implications

Tracking quality and deployment cost should be considered together when selecting an embedded pedestrian tracking configuration. Task-specific adaptation improved tracking without changing the YOLOv8n architecture or OC-SORT configuration. Its effect on throughput and EPF must still be considered.

The reported measurements may inform detector selection for applications that can tolerate low frame rates or intermittent tracking updates. However, they do not establish suitability for latency-sensitive applications that require conventional real-time video processing. Application suitability should be determined according to the required temporal resolution, tracking quality, and available computational resources.

### 5.2. Limitations

The findings should be interpreted within the scope of the evaluated configurations. The selected detectors represent distinct computational profiles but do not cover all available edge-oriented detector families, including YOLOv10, RT-DETR, and EfficientDet. All configurations were integrated with OC-SORT and evaluated using MOT17 on the NVIDIA Jetson Nano. Results may therefore differ for other detector families, trackers, benchmarks, or embedded platforms. Experiment 1 did not control input resolution across detectors. Consequently, it does not isolate detector architecture under a common input resolution.

The construction of the fine-tuning dataset introduced redundancy between the MOT16 and MOT17Det representations of the same training sequences. The dataset audit found no overlap with the MOT17 test sequences used for evaluation. This redundancy therefore does not constitute train–test leakage. However, it may have increased the relative contribution of repeated scenes during detector adaptation, and its effect on the reported results was not quantified.

Each adapted detector was trained once, and the deployment measurements were not obtained through controlled repeated trials. The reported comparisons therefore do not characterize variability arising from model initialization, data ordering, or system operation. Accordingly, the observed differences should be interpreted as descriptive results under the evaluated configurations rather than as statistical evidence of superiority.

RAM utilization and power consumption were measured at the system level. These measurements characterize total physical RAM occupancy and total board power consumption, but they do not isolate the resources used by the detector and OC-SORT. Detector inference, post-processing, data association, and track-state updates were also not timed separately. In addition, synchronized per-frame measurements linking pedestrian count, retained detections, active tracks, and pipeline latency were not collected. The present results therefore do not quantify component-specific resource consumption or the independent effect of scene density on deployment performance. A desktop-only idle baseline was not recorded. Consequently, the incremental board power and physical RAM utilization associated with the complete MOT pipeline relative to the idle system could not be calculated.

The evaluated pipelines processed between 0.4 and 5.0 FPS and did not achieve conventional real-time video rates. Inference was performed using FP32 PyTorch configurations without TensorRT conversion, FP16 or INT8 quantization, or structured pruning. The reported measurements therefore represent deployment baselines for the evaluated configurations rather than the upper performance limit of the Jetson Nano. Hardware-specific optimization may change throughput and EPF, but its effect requires direct experimental evaluation.

## 6. Conclusions

This study examined task-specific detector adaptation for MOT deployment on an NVIDIA Jetson Nano. Regarding the first research question, the evaluated configurations presented different trade-offs between tracking quality and deployment efficiency. Faster R-CNN achieved the highest tracking metrics, whereas SSD provided the highest throughput and lowest EPF. YOLOv8 provided an intermediate profile and was therefore selected for task-specific adaptation. These findings apply to complete deployment configurations because detector-specific preprocessing and inference resolutions were retained.

Regarding the second research question, the paired YOLOv8n comparison showed that MOTA increased by 35.14 percentage points. System-level RAM utilization and average board power consumption remained comparable. Within the evaluated configuration, these observations indicate that task-specific adaptation increased tracking performance while producing limited changes in the measured deployment characteristics.

The study provides an empirical assessment of detector adaptation using tracking and system-level deployment metrics. The findings apply to the evaluated detector-OC-SORT configurations, MOT17 benchmark, and NVIDIA Jetson Nano platform. Because the evaluated pipelines processed between 0.4 and 5.0 FPS, the reported results characterize PyTorch deployment baselines rather than conventional real-time MOT operation.

Future work will compare the detector architectures under a common input resolution. Detector training will be repeated using multiple random seeds and a deduplicated dataset. Deployment measurements will also be repeated under controlled conditions to report dispersion, confidence intervals and a desktop-only idle baseline. The evaluation will be extended to additional detector architectures, trackers, embedded platforms, and MOT benchmarks. Component-level timing and memory profiling will be combined with synchronized scene-density measurements. Hardware-specific optimization will be examined separately.

## Figures and Tables

**Table 1:** Summary of the experimental design adopted in this study.

Experiment	Purpose	Dataset	Evaluated MOT Pipelines
Experiment 1 Baseline Characterization and Detector Selection	Compare representative MOT Pipelines Select a detector for adaptation.	MOT17	YOLOv8, SSD, Faster R-CNN, All with OC-SORT
Experiment 2 Task-Specific Detector Adaptation	Evaluate task-specific detector adaptation under the same tracking and deployment protocol.	Merged Person Dataset (training) MOT17 (evaluation)	YOLOv8n, YOLOv8s, All with OC-SORT

**Table 2:** Detector and tracker confidence settings used during Jetson Nano inference.

Detector	Detector Confidence Threshold	Detector NMS IoU Threshold	OC-SORT Threshold	Implementation Details
YOLOv8n/ YOLOv8s	0.25	0.70	0.30	Ultralytics prediction defaults. Only the person class was retained. The same settings were used for baseline and adapted models.
SSDLite320	0.001	0.55	0.30	Torchvision detector defaults. Only predictions assigned to the person class were provided to OC-SORT.
FasterR-CNN	0.05	0.70 (RPN) 0.50 (final boxes)	0.30	Torchvision detector defaults. Only predictions assigned to the person class were provided to OC-SORT.

**Table 3:** Datasets employed in the experimental evaluation, including their role, annotation format, preparation procedure, and use throughout the proposed experiments.

Dataset	Role	Annotation Format	Preparation	Experiment
MOT17 [23]	Benchmark evaluation	MOTChallenge	Original annotations	Experiments 1 and 2
MOT16 [23]	Detector adaptation	MOTChallenge	Converted to COCO	Experiment 2
MOT20 [25]	Detector adaptation	MOTChallenge	Converted to COCO	Experiment 2
Person-mfa1g [26]	Detector adaptation	COCO	Merged without annotation changes	Experiment 2

**Table 4:** Deployment performance obtained during Experiment 1 according to the following metrics: Frames-per-Second (FPS), average Power Consumption, average system-level memory usage and estimated Energy per Frame (EPF).

Detector	FPS	Power (mW)	System RAM Used (GB)	EPF (mJ/Frame)
YOLOv8n	3.6	4235	3.3820	1176.39
Faster R-CNN	0.4	4252	3.3821	10,630.00
SSD	5.0	4201	3.3625	840.20

**Table 5:** Deployment performance obtained during Experiment 2 according to the following metrics: Frames-per-Second (FPS), average Power Consumption, average system-level memory usage and estimated Energy Per Frame (EPF).

Detector	FPS	Power (mW)	System RAM Used (GB)	EPF (mJ/Frame)
Baseline YOLOv8n	3.60	4235	3.3820	1176.39
Task-Specific YOLOv8n	3.50	4227	3.3925	1207.71
Task-Specific YOLOv8s	2.50	4221	3.4091	1688.40

## Data Availability

All datasets used in this study are publicly available from their original sources. MOT17, MOT16 [23], and MOT20 [25] are available through the MOTChallenge benchmark, while the Person-mfa1g [26] dataset is publicly available from its original repository. Table 3 summarizes the datasets employed in this study. The source code developed for the experiments is available from the corresponding author upon reasonable request.
